# The Role of GPX Enzymes, Lipid Profiles, and Iron Accumulation in Necrotizing Enterocolitis

**DOI:** 10.3390/ijms26136077

**Published:** 2025-06-25

**Authors:** Grant H. Gershner, Chase Calkins, Alena Golubkova, Camille Schlegel, Aslan Massahi, Megan Lerner, Alex N. Frickenstein, Sarah Bonvicino, Martin-Paul Agbaga, Catherine J. Hunter

**Affiliations:** 1 Division of Pediatric Surgery, Department of Surgery, University of Oklahoma Health Sciences Center, Oklahoma City, OK 73104, USA; grant-gershner@ouhsc.edu (G.H.G.);; 2 Department of Surgery, The University of Oklahoma Health Sciences Center, 800 Research Parkway, Suite 449, Oklahoma City, OK 73104, USA; megan-lerner@ouhsc.edu; 3 Stephenson School of Biomedical Engineering, University of Oklahoma, Norman, OK 73019, USA; 4 Lipid Analysis Core, Department of Ophthalmology, Dean McGee Eye Institute, University of Oklahoma Health Sciences Center, Oklahoma City, OK 73104, USA

**Keywords:** ferroptosis, iron, necrotizing enterocolitis, lipidomics, glutathione peroxidase, oxidative stress

## Abstract

Necrotizing enterocolitis (NEC) is a serious GI disease of premature infants, marked by intestinal inflammation and necrosis. Recent research has highlighted the potential role of oxidative stress (OS) and ferroptosis in its pathogenesis. We previously identified a deficiency in Glutathione Peroxidase (GPX) 4 and lipid radical accumulation, prompting further investigation. Human intestinal tissue from a prior study was processed, and it underwent RNA and protein isolation, Immunohistochemistry, Immunofluorescence, and acid digestion for iron and selenium analysis via Inductively coupled mass spectrometry (ICP-MS). NEC was induced in human enteroids using lipopolysaccharide (LPS) and hypoxia, followed by RNA/protein isolation and lipidomic analysis. Humans with NEC had significantly higher levels of GPX2 (*p* = 0.0003). Enteroids exposed to NEC conditions had significantly decreased amounts of NADPH compared to initial controls (*p* = 0.0091), but similar levels compared to post-24 h controls (*p* = 0.3520). Patients with NEC had significantly higher levels of iron compared to controls via the bathophenanthroline-based assay (*p* = 0.0102) and with ICP-MS (*p* = 0.0148). There were several significant alterations in lipid distribution between NEC and control patients, but not in the fatty acid profiles. Our study suggests that oxidative stress, iron dysregulation, and altered lipid metabolism contribute to NEC pathogenesis.

## 1. Introduction

Necrotizing enterocolitis (NEC) is a severe gastrointestinal disorder predominantly affecting premature neonates. It is characterized by intestinal inflammation and subsequent necrosis. Epidemiologically, NEC affects between 7–10% of very low birth weight (VLBW) infants (<1500 g), with its incidence inversely correlated with gestational age and birth weight [1]. Risk factors include prematurity, formula diet, and hypoxia. NEC remains a major cause of morbidity and mortality in neonatal intensive care units (NICUs), with overall mortality rates ranging from 20–30%, increasing significantly in cases that require surgical intervention. Despite years of expansive research, NEC remains a prominent problem, resulting in 1 in 10 of all neonatal deaths, and the primary cause has yet to be identified [2]. The pathogenesis has been determined to be multifactorial. Some factors include intestinal immaturity, aberrant immune responses, and an inability to dampen a hyperinflammatory state [3,4]. These lead to a “leaky gut”—a state of epithelial barrier dysfunction. This, in combination with intestinal dysbiosis, leads to increased pathogen translocation and subsequent sepsis [5].

A recent area of interest for NEC research has been the role of oxidative stress (OS). OS is a known cause of long-term morbidity in premature infants. This is seen in the form of retinopathy of prematurity (ROP), bronchopulmonary dysplasia (BPD), periventricular leukomalacia (PVL), and others [6]. The role of OS in the pathogenesis of NEC is through its impact on immature intestinal tissues. In NEC, the imbalance between pro-oxidant and antioxidant systems leads to excessive reactive oxygen species (ROS) production, which contributes to cellular damage and inflammation [7]. Premature neonates are especially vulnerable to oxidative damage due to underdeveloped antioxidant defense mechanisms [8]. This oxidative burden can induce intracellular modifications, such as protein and lipid peroxidation.

Ferroptosis is a novel form of regulated cell death that differs from other forms of programmed cell death, like apoptosis or pyroptosis. Ferroptosis is characterized by (1) a deficient or ineffective innate antioxidant system (namely glutathione (GSH) system), (2) accumulation of redox-active iron, and (3) accumulation of oxidized polyunsaturated fatty acids (PUFAs) [9]. Previous works by our lab found that the GSH antioxidant system is more oxidized in NEC, indicating OS. We also examined levels of a key regulatory enzyme of OS, glutathione peroxidase 4 (GPX4). Through PCR and Western Blotting, we were able to demonstrate that GPX4 levels were significantly decreased in NEC. In the same study, we found that NEC was associated with evidence of lipid peroxidation in the form of the lipid aldehydes 4-hdroxy-2-nonenal (4-HNE) and 4-hydroxy-2-hexenal (4-HHE) [10].

While GPX4 is a key enzyme of ferroptosis, it is part of a family of other GPX proteins. GPX1 is found systemically. Its main targets in OS are hydrogen peroxide (H_2_O_2_) and soluble low-molecular hydroperoxides. While GPX4 is able to target more complex lipid-hydroperoxides, GPX1 cannot, limiting its ability to prevent ferroptosis. GPX2 is mostly found in gastrointestinal and hepatic tissues. It is mainly found in the crypts of the colon or the villi of the small intestine. Here, it acts as the first barrier to hydroperoxides that are generated from food. Additionally, through WNT signaling, GPX2 plays an important role in epithelial homeostasis of the intestine [11].

Although it is distinct from the GPX system, NADPH Oxidases (NOX) enzyme family cross talk GPX enzymes through NADPH in the setting of oxidative stress. NOXs are transmembrane proteins that use NADPH to transport electrons across membranes in order to generate superoxides [12]. Common traits included six conserved transmembrane domains, NADPH-binding sites, a FAD-binding region, and four conserved histidines [13]. Members of this enzyme family include NOX 1, 2, 3, 4, and 5, as well as Dual Oxidase 1 and 2 (DUOX1 or 2). In order to keep these enzymes in check, they require several factors before they are fully activated. For NOX 1, 2, and 3, this is NADPH oxidase organizer 1 (NOXO1), NADPH Oxidase-activating protein 1 (NOXA1), and Rac1 [14].

DUOX is similar to NOX enzymes, except for the addition of an intracellular region and a seventh transmembrane region. DUOX activity requires maturation factors in order to generate ROS. These are DUOXA1 and DUOXA2, and they regulate DUOX expression by controlling ER-to-Golgi transition of mature DUOX enzymes, as well as organizing elements for their surface expression [13,15]. The role of DUOX1 and 2 has been implicated in a variety of OS-based pathologies, comprising Alzheimer’s disease, cervical cancer, and inflammatory bowel disease [16,17,18].

We have identified evidence of a role for ferroptosis in NEC on the basis of a deficient GSH system and elevated lipid radicals. Therefore, we sought to further elucidate the role ferroptosis plays in NEC. To expand on our research, we utilize human intestinal tissue and intestinal organoids to quantify tissue iron levels, perform lipid and fatty acid profiling, evaluate the inflammatory milieu, and examine other aspects of oxidative stress in NEC. We hypothesize that NEC would be associated with intestinal iron accumulation and a decrease in GPX 1 and 2. Furthermore, we anticipated an increase in oxidative stress, as demonstrated by decreased NADPH levels and alterations in lipid and fatty acid profiles.

## 2. Results

### 2.1. GPX1, 2, and GSS DNA Expression

**Figure 1:**
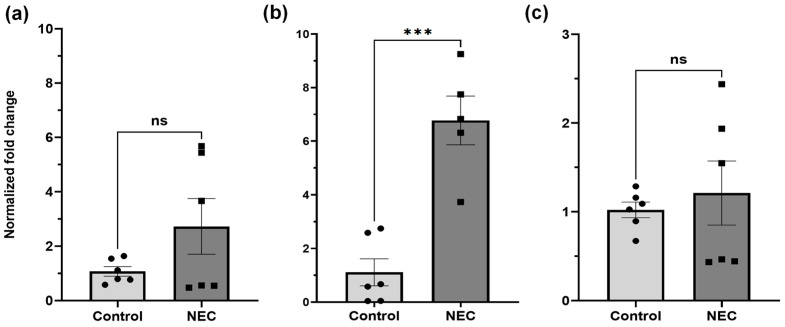
Expression of GPX1, GPX2, and GSS via RNA RTqPCR. Expression of (**a**) GPX1 and (**b**) GSS were not significantly different between active NEC and control patients (*p* > 0.05). GPX2 (**c**) was significantly elevated in patients with active NEC when compared to controls (*p* < 0.05). Asterisk indicate statistical significance, ns indicates no significance. Error bars represent the standard error of the mean (SEM).

Age-matched human intestinal samples showed no statistical difference the between control and NEC sample mRNA for GPX1 (*p* = 0.1431, −0.664–1.04). Tissue samples from patients with NEC had significantly elevated levels of GPX2 mRNA compared to control samples (*p* = 0.0003, 6.77–1.82). There was no significant difference in the level of GSS mRNA between NEC and control tissue samples (*p* = 0.6212; 1.21–0.81) (Figure 1).

### 2.2. GPX2 Immunofluorescence and Immunohistochemistry

**Figure 2:**
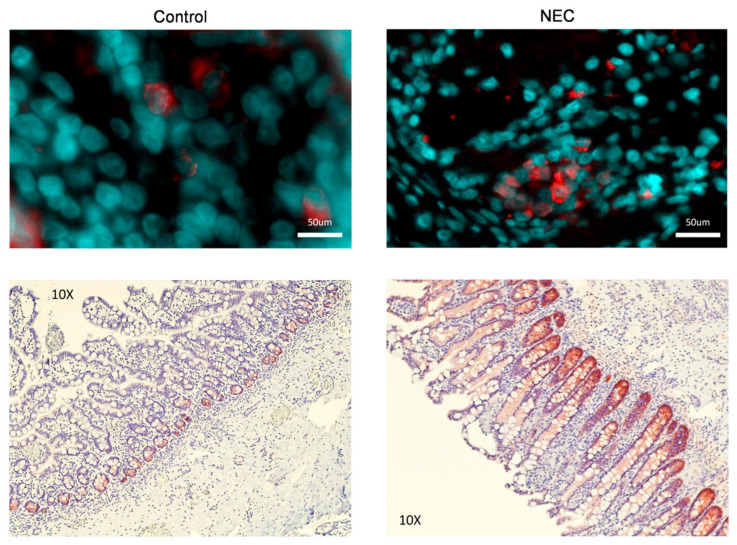
Immunofluorescent and Immunohistochemical Staining of Neonatal Intestine for GPX2. Microscopic examination of human neonatal intestine. GPX2 appears red while nuclear components are counterstained with DAPI blue. Additionally, microscopic histological images of the neonatal intestine are shown. In these images, GPX appears red and all other cell components are purple/blue. We noted increased staining of GPX2 in both experiments in tissue from those with active NEC.

Immunofluorescent (IF) and Immunohistochemistry (IHC) staining of tissues showed increased amounts of GPX2 staining in the NEC tissue vs. control tissue (Figure 2).

### 2.3. NADPH Concentration, and ReDox Gene RNA-Sequencing

**Figure 3:**
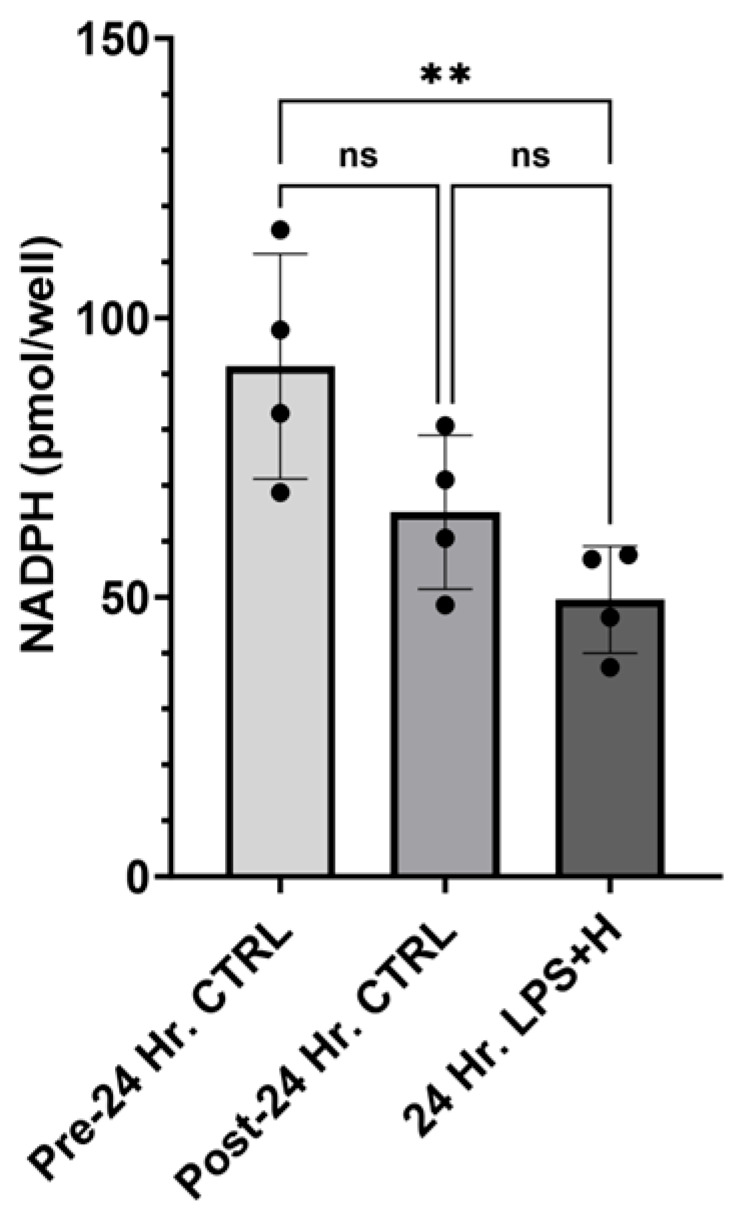
NADPH Concentrations in Enteroids Exposed to NEC Conditions. NADPH concentrations were measured prior to experimentation in control enteroids. One group was kept as controls, while another was subjected to NEC induction for 24 h. There was a significant drop in NADPH concentrations between the initial control and the NEC-induced group (*p* < 0.05). There was no significant difference between the initial control and the 24 h control (*p* > 0.05), or the 24 h control and the NEC induction group (*p* > 0.05). Asterisk indicate statistical significance, ns indicates no significance. Error bars represent the standard error of the mean (SEM).

Utilizing our enteroid NEC model, we performed a NADPH assay. There was a trend toward decreased levels of NADPH in enteroids maintained in control conditions after 24 h, but this change was not significant (*p* = 0.087, −3.78–56.0). Enteroids exposed to 24 h of hypoxia and LPS had significantly decreased amounts of NADPH compared to the initial measurements (*p* = 0.0091, 11.87–71.62), but similar levels compared to enteroids maintained in control conditions for 24 h (*p* = 0.3520, −14.22–45.53; Figure 3). Statistical analysis was performed via One-Way ANOVA.

**Figure 4:**
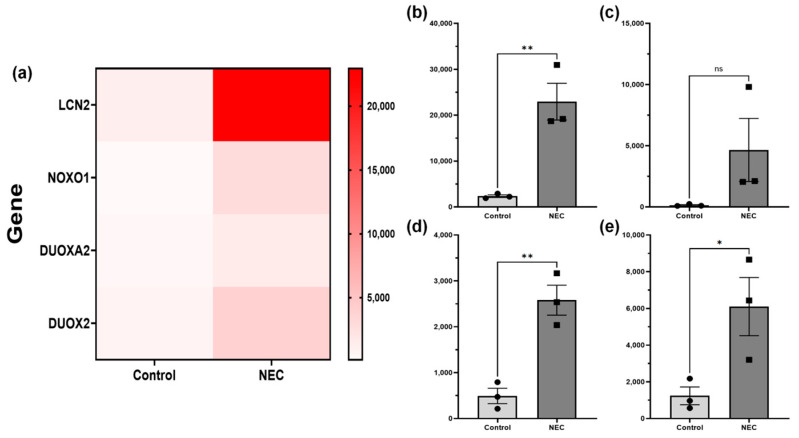
RNA-Sequencing Heatmap and Quantitative Expression of Common Redox Proteins. RNA-sequencing (**a**) found significantly higher levels of (**b**) LCN2, (**d**) DUOXA2, and (**e**) DUOX2 (*p* < 0.05). There was no significant difference in (**c**) NOXO1 (*p* = 0.154) when comparing enteroids from patients with active NEC to control patient enteroids. Asterisk indicate statistical significance, ns indicates no significance. Error bars represent the standard error of the mean (SEM).

When comparing RNA-sequencing of enteroids from patients with active NEC, who had recovered from NEC, and control patients, a total of 60,610 genes were compiled into a library. We found that the NOXO1 (*p* = 0.0001, −2635–11,670), DUOX2 (*p* = 0.043, 260–9463), and DUOXA2 (*p* = 0.0047, 1068–3107) were significantly upregulated in enteroids from patients with NEC compared to control patient enteroids (Figure 4). Additonally, the proinflammatory and pro-ferroptotic enzyme LCN2 was also significantly elevated in NEC (*p* = 0.007, 9433–31,760). RNA-Seq data were not generated for GPX1, 2, or 4.

### 2.4. NEC Increases Intestinal Iron Concentration and Is Not Deficient in Selenium

**Figure 5:**
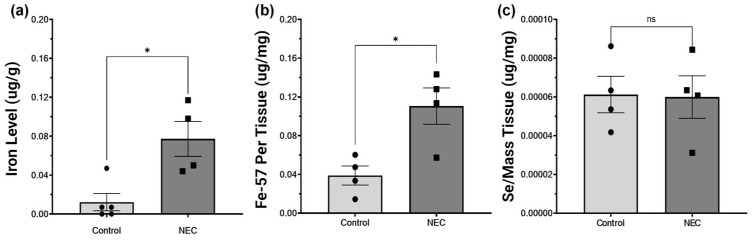
Tissue Iron and Selenium Levels by Colorimetric and Inductively Coupled Plasma Mass Spectrometry. In tissue from active NEC patients and control patients, iron levels were significantly elevated in both the bathophenanthroline colorimetric assay (**a**), as well as when compared using ICP-MS ((**b**), *p* < 0.05). Tissue selenium levels were not significantly different ((**c**), *p* > 0.05). Asterisk indicate statistical significance, ns indicates no significance. Error bars represent the standard error of the mean (SEM).

Tissue samples fom patients with NEC had significantly higher levels of iron compared to control tissue samples when examined with the bathophenanthroline-based assay (*p* = 0.01, 0.02091–0.1092). This was subsequently confirmed on ICP-MS (*p* = 0.015, 0.1105–0.0325) (Figure 5a,b).

Given that GPX enzymes are selenoproteins, we sought to assess selenium levels as a possible explanation for this deficency. However, the tissue from patients with NEC had no difference in levels of selenium compared to control tissue samples when compared through ICP-MS (*p* = 0.932, −3.662 × 10^−5^–3.404 × 10^−5^ (Figure 5c).

### 2.5. Lipids Profiling

**Figure 6:**
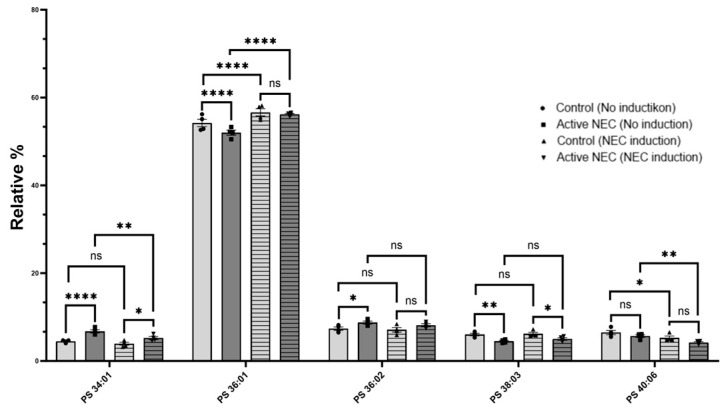
Expression of Phosphatidylserines in Control Versus Active NEC Enteroids Before and After NEC Induction. Lipidomic analysis of PS from control and active NEC enteroids before and after NEC induction. Included are PS 34:01, 36:01, 36:02, 38:03, and 40:06 for their significant differences. Asterisk indicate statistical significance, ns indicates no significance. Error bars represent the standard error of the mean (SEM).

Phosphatidylserine (PS) 34:01 was significantly elevated in patients with active NEC (*p* < 0.0001), but did not increase after NEC induction (*p* < 0.005). PS 36:01 was decreased in active NEC enteroids (*p* < 0.0001) but significantly increased after NEC induction (*p* < 0.0001) to similar levels as controls (*p* = 0.741). There was also a significant increase in control patient levels after NEC induction (*p* < 0.0001). PS 40:06 had similar levels between the control and active NEC patients (*p* = 0.286). There was a significant decrease in both control (*p* = 0.040) and NEC (0.0084) enteroids after NEC induction, though there was no significant difference between the two (*p* = 0.0980). Other PSs found include PS 36:02, 38:02, 38:03, 38:04, 40:01, 40:04, 40:05, and 42:01. There was no statistically significant difference between control or active NEC enteroids, or those that underwent NEC induction (Figure 6).

**Figure 7:**
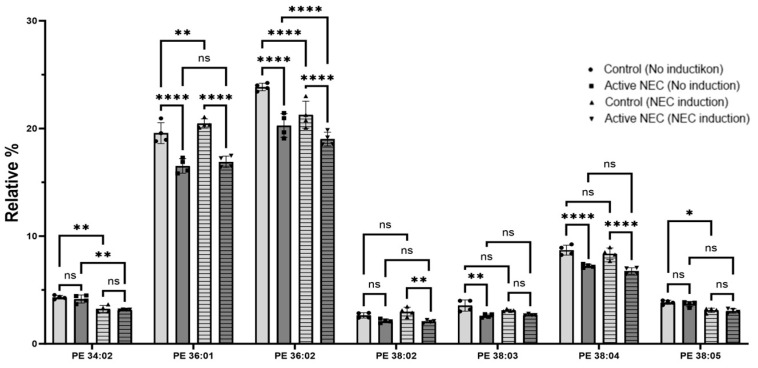
Expression of Phosphatidylethanolamine in Control Versus Active NEC Enteroids Before and After NEC Induction. Lipidomic analysis of PE from enteroids from control and active NEC patients that subsequently underwent NEC induction. Included are PE 34:02, 36:01, 36:02, 38:02, 38:03, 38:04, and 38:05 for their significant differences. Asterisk indicate statistical significance, ns indicates no significance. Error bars represent the standard error of the mean (SEM).

Phosphatidylethanolamine (PE) 34:02 levels were similar between control and NEC patients at baseline (*p* = 00.935) and after NEC induction (*p* = 0.989). There was a significant drop in levels after NEC induction for both control (*p* = 0.001) and active NEC patients (*p* = 0.0030). PE 36:01 was significantly decreased in active NEC patients before and after induction (*p* < 0.0001 for both). There was no significant change in levels after NEC induction (*p* = 0.552). PE 36:02 was significantly decreased in active NEC enteroids before and after induction (*p* < 0.0001 for both). There was a significant decrease in control and active NEC enteroid levels after induction (*p* < 0.0001 for both). PE 38:04 was significantly decreased in active NEC patients before and after NEC induction (*p* < 0.0001 for both). There was no significant change in control or NEC enteroid levels after induction (*p* = 0.636 and *p* = 0.365, respectively). Other PE identified include 32:01, 34:01, 36:03, 36:04, 38:02, 38:03, 38:04, 38:5, 40:05, and 40:06. There was no statistically significant difference between control or active NEC enteroids, or those that underwent NEC induction (Figure 7).

**Figure 8:**
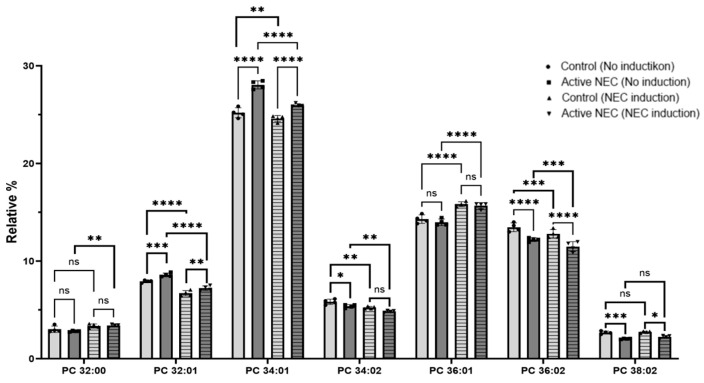
Expression of Phosphatidylcholines in Control Versus Active NEC Enteroids Before and After NEC Induction. Lipidomic analysis of PC from enteroids from control and active NEC patients that subsequently underwent NEC induction. Included are PC 32:00, 32:01, 34:01, 34:02, 36:01, 36:02, and 38:02 for their significant differences. Asterisk indicate statistical significance, ns indicates no significance. Error bars represent the standard error of the mean (SEM).

Phosphatidylcholine (PC) 32:00 had a significant increase in levels before and after NEC induction for both active NEC enteroids (*p* = 0.0037), but not for controls (*p* = 0.1762). There were similar levels between active NEC and control patients before and after induction (*p* = 0.637 and *p* = 0.996, respectively). There were significantly higher levels of PC 32:01 in active NEC patients before (*p* = 0.0005) and after (*p* = 0.0085) NEC induction. There was a significant drop in levels for both control and active NEC enteroids after induction (*p* < 0.0001). PC 34:01 was significantly elevated in active NEC enteroids before and after induction (*p* < 0.001 for both). There was a significant decrease in levels for both control and active NEC enteroids after NEC induction (*p* = 0.0025 and *p* < 0.0001, respectively). PC 34:02 was significantly decreased in active NEC patients before, but not after, NEC induction compared to controls (*p* = 0.0370 and *p* = 0.0951, respectively). There was a significant decrease in levels for both control and active NEC enteroids after induction (*p* = 0.0027 and *p* = 0.0094, respectively). PC 36:01 had similar levels between control and NEC enteroids before and after NEC induction (*p* = 0.216 and *p* = 0.781, respectively). There was a significant increase in levels of this lipid after induction for the control and NEC cell lines (*p* < 0.0001, respectively). PC 36:02 was significantly decreased between control and active NEC enteroids at baseline and after NEC induction (*p* < 0.0001 for both). There were significant decreases in levels of this lipid for control and active NEC enteroids after induction (*p* = 0.0004 and *p* = 0.0002, respectively). PC 38:02 was significantly decreased between active NEC and control enteroids before and after induction (*p* = 0.0009 and *p* = 0.015, respectively). There was no significant change after induction for control and NEC cell lines (*p* = 0.984 and *p* = 0.646, respectively). Finally, the sum of ether-PC was significantly elevated in active NEC enteroids compared to controls, regardless of NEC induction (*p* < 0.0001 for both). There was no significant increase among control enteroids (*p* = 0.599), but there was a significant increase among active NEC enteroids (*p* = 0.0002). Other PCs identified include 30:00, 30:01, 30:02, 36:03, 36:04, 38:01, 38:03, 38:04, and 38:05. There was no statistically significant difference between control or active NEC enteroids, or those that underwent NEC induction (Figure 8).

In summary, we found several significant alterations in PSs, PEs, and PCs in enteroids from patients with active NEC compared to control patient enteroids. Additionally, we found several significant alterations in enteroids from both control and active NEC patients after underoing our NEC model.

### 2.6. Profile of Plasmalogen-Containing Lipids

**Figure 9:**
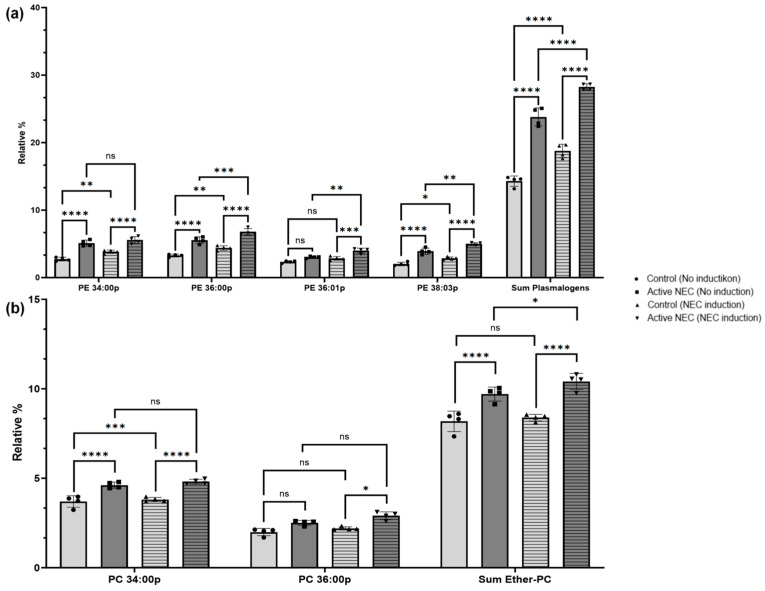
Expression of Plasmalogens and Ether-Phosphatidylcholines in Control Versus Active NEC Enteroids Before and After NEC Induction. Lipidomic analysis of (**a**) plasmalogens and (**b**) ether-PC from enteroids from control and active NEC patients that subsequently underwent NEC induction. (**a**) Included PE-plasmalogens are PE 34:00 p, 36:00 p, 36:01 p, 38:03 p, and the sum of all plasmalogens for their significant differences. (**b**) Included ether-PC include PC 34:00 p, 36:00 p, and the sum of ether-PCs for their significant differences. Asterisk indicate statistical significance, ns indicates no significance. Error bars represent the standard error of the mean (SEM).

PE-containing plasmalogen, 34:00 p, was significantly elevated in active NEC patients compared to controls (*p* < 0.0001). After NEC induction, this elevation persisted (*p* < 0.0001), but not significantly (*p* = 0.375). There was a significant increase in PE 34:0 *p* in the control line after NEC induction (*p* = 0.001). Similarly, PE 36:00 p was significantly elevated in active NEC enteroids before and after NEC induction (*p* < 0.0001 for both). There was a significant increase in the levels for both control and NEC enteroids after NEC induction (*p* = 0.0007 and *p* = 0.0001, respectively; Figure 9a).

PE 36:01 p-levels were similar at baseline (*p* = 0.076) and significantly increased after NEC induction (*p* = 0.0004) between the control and active NEC patients. There was no significant change after NEC induction in control patients (*p* = 0.262), but there was a significant increase in active NEC enteroids (*p* = 0.004; Figure 9a).

PE 38:03 p was significantly elevated in active NEC enteroids at baseline and after NEC induction compared to controls (*p* < 0.0001). There was a significant increase in levels after NEC induction for both control and active NEC patients (*p* = 0.016 and *p* = 0.0013, respectively). Finally, the sum of plasmalogens in active NEC enteroids was higher before and after NEC induction (*p* < 0.0001 for both). There were significant increases in plasmalogen levels in control and NEC enteroids after induction (*p* < 0.0001 for both; Figure 9a).

PC 34:00 p was significantly elevated in active NEC enteroids before and after NEC induction (*p* < 0.0001). There was not a significant increase amongst the groups (*p* = 0.912 and *p* = 0.646, respectively). PC 36:00 p was significantly elevated in active NEC enteroids before and after induction compared to controls (*p* = 0.0113 and *p* = 0.0002, respectively). There was no significant change in levels amongst the groups after NEC induction (*p* = 0.608 and *p* = 0.089, respectively (Figure 9b).

### 2.7. Changes in Fatty Acid Composition

**Figure 10:**
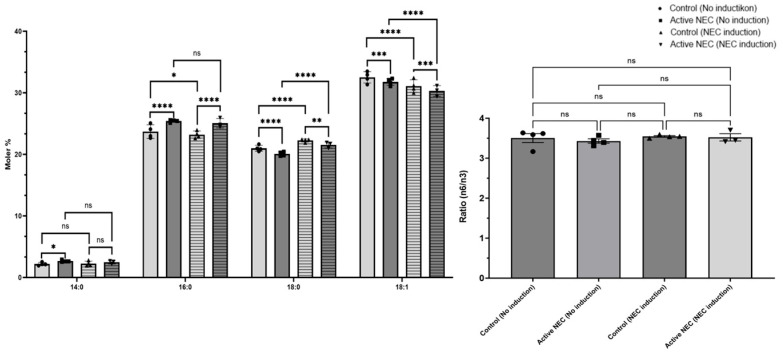
Expression of n6/n3 Ratios in Control Versus Active NEC Enteroids Before and After NEC Induction. Lipidomic analysis of FA and n6/n3 ratios from enteroids from control and active NEC patients that subsequently underwent NEC induction. Included are FA 14:0, 16:0, 18:0, and 18:1 for their significant differences. N6/N3 ratios are included, but not significant. Asterisk indicate statistical significance, ns indicates no significance. Error bars represent the standard error of the mean (SEM).

The fatty acids that were significantly altered between enteroids from patients with NEC compared to control patient enteroids include 16:0, 18:0, and 18:1. We did not observe any significant differences in the levels of polyunsaturated fatty acids, such as 20:4n6 and 22:6n3. At baseline, there were significantly higher levels of fatty acid (FAs) 14:0 in enteroids from patients with NEC compared to controls (*p* = 0.0489). FA 16:0 was significantly elevated at baseline in enteroids from active NEC patients (*p* < 0.0001), which persisted in enteroids that underwent NEC induction (*p* < 0.0001). There was a significant decrease of 16:0 in control enteroids after NEC induction (*p* = 0.0422), but not for enteroids from active NEC patients (*p* = 0.3341). On the other hand, FA 18:0 was significantly decreased in enteroids from NEC patients at baseline (*p* < 0.0001), as well as after NEC induction (*p* < 0.0001). There was a significant increase in this FA for both control and NEC patient enteroids after NEC induction (*p* < 0.0001 for both). FA 18:1 was significantly decreased in NEC patient enteroids at baseline (*p* = 0.0004). This difference persisted after NEC induction (*p* = 0.0006). There were significant decreases in both control and NEC patient enteroids after NEC induction (*p* < 0.0001 for both). Other FAs identified that showed no significant differences include 16:1, 18:2n6, 20:0, 20:1, 20:5n3, 20:2n6, 20:3n6, 20:4n6, 22:0, 22:1, 22:4n6, 22:5n3, 22:6n3, 24:0, 24:1, 26:0, 28:0, and 30:0. There was no statistically significant difference in the n6/n3 ratio between control and NEC patient enteroids prior to induction (*p* = 0.898) or after NEC induction (*p* = 0.997). There was no significant change from before and after induction for either cell line (*p* = 0.976 control, *p* = 0.848 NEC; Figure 10).

## 3. Discussion

In this study, we sought to explore the role of ferroptosis in NEC. We did so by examining other forms of the key enzymes in OS pathways (GPX, GSS, NADPH, DUOX, LCN), iron levels in the tissue from patients with and without NEC, and lipid profiles of enteroids from patients with and without NEC. We hypothesized that GPX 1, 2, and GSS would be deficient in NEC and that NEC patients would have higher levels of intestinal iron compared to controls. We also anticipated that enzymes associated with oxidative stress would be elevated in NEC, that there would be lower levels of available NADPH, and there would be alterations in lipid profiling. With the information gathered from the above experiments, we postulate that ferroptosis plays a significant role in the pathogenesis of NEC.

As previously stated, GPX4 is one of the key regulators of ferroptosis due to its ability to counteract lipid peroxidation [19]. While several studies have examined GPX1 and 2 as part of the signature of ferroptosis, the majority of findings are limited to adult oncologic studies [20,21,22]. While we did not find any significant changes in GPX1 and GSS expression, we did find a significant elevation in GPX2 expression in NEC patients. This was counter to our hypothesis. GPX2 is typically localized to the intestine and GI tract, where it is thought to act as a barrier against hydroperoxide absorption [23]. GPX2 downregulation was associated with increased apoptosis in intestinal crypts [24], while upregulation was found to lead to malignant progression, cisplatin resistance, and worse outcomes in various cancers [25]. A study by Steven et al. found that GPX 1 and 2 knockout mice that were selenium-deficient had increased severity of colitis in double knockouts. When mice were GPX2^+/−^, the incidence and severity of inflammation were reduced [26]. Additionally, a study by Tian et al. found that when GPX2 was knocked down, levels of lipid ROS and iron levels increased. The exact function of the upregulation we found in NEC is yet to be elucidated. Given the deficiency in GPX4, this could be the enteric cell’s response to somewhat counteract the progressive oxidative stress of NEC.

NADPH serves a dynamic role and is implicated in numerous cellular functions, including cellular metabolism, oxidative stress, antioxidant processes, immune functions, and cellular death [27]. The role of NADPH is paradoxical. On one hand, it serves as a substrate to generate ROS. On the other, it serves as the substrate for reduction of ROS. Therefore, we sought to quantify the total amount of NADPH within the cell. We found no statistical change in the concentration between 24 h of incubation and NEC induction.

At baseline, NOX proteins are involved in normal cellular processes within the cell; however, it has been shown that the aberrant function of these proteins is implicated in numerous diseases [12]. It is also of note that NOX proteins use the substrate NADPH to generate ROS. GPX4 also uses NADPH as a substrate. We have previously shown that GPX4, a known reducer of lipid radicals generated by ROS, is downregulated in NEC [10]. This lays the framework for our central hypothesis that ROS-driven generation of lipid radicals, coupled with the inability to combat this increase in oxidative stress, leads to ferroptosis and cellular death in the NEC-diseased neonatal intestine. LCN2 is similarly involved in numerous cellular processes, but most notably in inflammation, oxidative stress response, iron homeostasis, and Fenton reaction within the intestines [28].

**Figure 11:**
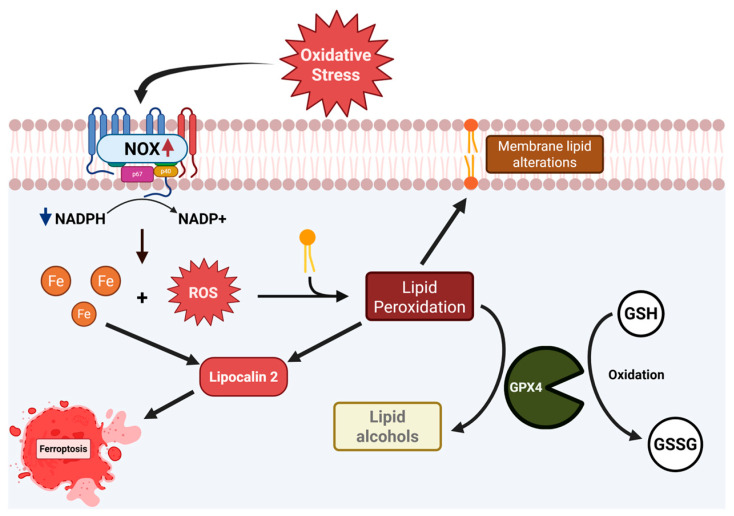
Pictogram Illustrating the Potential Role of Oxidative Stress and Ferroptosis in NEC. A diagram that illustrates oxidative stress and its role in the formation of lipid radicals and iron redistribution, as well as the interplay between NADPH Oxidases and Glutathione peroxidase enzyme families.

Iron dysregulation emerged as a significant feature of NEC, with patients exhibiting elevated iron levels confirmed through both the bathophenanthroline assay and ICP-MS. Excess iron can amplify oxidative stress through the Fenton reaction, where iron catalyzes the conversion of hydrogen peroxide into highly reactive hydroxyl radicals [29]. These radicals cause extensive damage to cellular components, including lipids, proteins, and DNA [30]. Our findings and the accompanying lipid remodeling observed in NEC further underscore the pathological role of iron dysregulation. Elevated levels of specific PS, PE, and PCs, alongside alterations in fatty acid profiles, suggest iron-induced oxidative modifications to membrane lipids. These changes compromise membrane integrity and disrupt cellular signaling [31,32]. The interplay between iron-catalyzed oxidative stress, lipid remodeling, and the generation of bioactive inflammatory mediators highlights a critical axis in NEC pathology, offering potential targets for therapeutic intervention. These could be aimed at mitigating iron overload, reducing oxidative stress, and preserving membrane structure and function. This interplay is demonstrated in Figure 11.

We have previously shown that by-products of lipid peroxidation accumulate in NEC [10]. Our previous experiment examined human tissue from control patients and those with active NEC. In this study, we expanded on that by using enteroids from control patients and patients with active NEC. From this, we found several significant differences in lipid composition among the PC, PS, and PE. Amongst PS, NEC enteroids were found to have significantly higher levels of plasmalogens. These phospholipids contain a *cis* double bond conjugated with the ether oxygen at the sn-1 position [33,34]. These normally contribute to cell membranes as lipid rafts that contain proteins used in cell signaling, cell–cell interactions, and endocytosis [34]. Plasmalogens have also been suggested as possible “sacrificial oxidants.” The hydrogen atoms adjacent to their vinyl ether bond have low dissociation energies, making them an easier target for oxidation [35]. This sacrificial behavior has been shown to slow or halt lipid oxidation propagation [36]. Yet, a recent study by Nyström et al. examined mucosal biopsies from pediatric patients with inflammatory bowel disease (IBD). In active IBD, ileal mucosa biopsies had elevated levels of plasmalogens, which makes them more susceptible to inflammation [37]. However, several studies found that oxidative stress was associated with decreased levels of plasmalogens in disease states like lupus, Gaucher disease, and sepsis [38,39,40]. One possible explanation for this paradoxical behavior is the body attempting to increase plasmalogens to staunch the propagation of ferroptosis.

While this behavior would limit the propagation of lipid peroxidation, plasmalogens have been shown to function as a sink for polyunsaturated fatty acids (PUFAs) [41]. PUFAs are a group of fatty acids that contain more than one double bond in their lipid chain [42]. PUFAs are mediators of inflammation, especially those of the n-6 PUFAs. This group of PUFAs feeds into the eicosanoid family of inflammatory mediators, like thromboxanes and leukotrienes [43]. In our previous study, we found elevated n6/n3 ratios, which corresponded to significantly elevated levels of 4-HHE (a product of n-3 PUFA peroxidation) and decreased 4-HNE (a product of n-6 PUFA peroxidation). We did not find any differences in the n6/n3 ratios when we examined the fatty acids. This could be because we performed the experiments on cultured enteroids instead of native tissue. The enteroids in both groups are exposed to the same levels of fatty acid precursors. In this case, patients would have varying amounts of n6 and n3 exposure, depending on their diet [44].

## 4. Materials and Methods

### 4.1. Human Intestinal Sample Collection

Institutional Review Board approval (#11610-11611) was obtained before the commencement of human tissue collection. After parental consent, a section of intestinal tissue was collected from neonates and infants undergoing clinically indicated bowel resections at the Oklahoma Children’s Hospital (Oklahoma City, OK, USA). Tissue was resected in cases of Bell Stage 3 NEC (surgical NEC) or for non-inflammatory surgical cases such as congenital gastrointestinal malformations (i.e., gastroschisis strictures, intestinal atresias, etc.). The latter were used as the control tissue for comparison. Gestational age at birth, age at surgery (corrected estimated gestational age—cEGA), type of sample (ileum, jejunum, etc.), sex, and indications for surgical resection were noted for each sample included. All intestinal samples were sectioned, washed in cold Dulbecco’s phosphate-buffered saline (DPBS, Sigma Life Science, #D8573, St. Louis, MO, USA), and either snap-frozen in dry ice before further processing or stored in Roswell Park Memorial Institute media (RPMI, Gibco, #11875-093, Waltham, MA, USA) for less than 24 h to be processed into enteroids.

### 4.2. Human Neonatal Enteroid Cultures

When enteroids are generated from intestinal samples, they are made within 24 h of tissue collection. Intestinal crypt isolation and enteroid processing were completed based on our previously described protocol [45]. Following processing, enteroids were suspended in basement membrane matrix domes (BMM, Corning, #CB-40230C, Corning, NY, USA). They were maintained using 50% LRWN-conditioned media. This is supplemented with 1 mM N-Acetylcysteine (Millipore Sigma, #A9165-5G, Burlington, MA, USA), 500 nM A-83-01 (R&D Systems, #2939/10, Minneapolis, MN, USA), 10 µM SB202190 (Millipore Sigma, #S7067-5 MG, Burlington, MA, USA), 10 mM Nicotinamide (Millipore Sigma, #N0636-100G, Burlington, MA, USA), and 10 nM [leu] 15-gastrin 1 (Millipore Sigma, #G9145-0.1 MG). Enteroids were passaged every 5–10 days, depending on microscopic appearance. These were experimented on once they were deemed mature (between passages 4–10, signs of budding microscopically) for all experiments.

Similar to our lab’s collection of intestinal samples, we also created human intestinal organoids (enteroids). Under the same IRB approval we have a collection of enteroids. Enteroids were harvested as above and passaged until mature. Once mature, these were processed similarly to passaging and suspended in 500 µL of CTS^TM^ Synth-a-freeze^TM^ Media (Gibco, #A13713-01, Waltham, MA, USA). This was then stored at −80 °C until Mycoplasma testing could take place (R&D Systems, #CUL001B, Minneapolis, MN, USA). Once negative, frozen enteroids were transferred to liquid nitrogen (−150 °C) for long-term storage.

To revive frozen enteroids, cryovials are removed from the liquid nitrogen and moved to dry ice until ready. Then, 5 mL of Fernando Wash Medium (FWM, 43.5 mL DMEM/F12, 500 µL 1M HEPES, 500 µL penicillin/streptomycin, 500 µL of 100× glutaMAX, and 5 mL neonatal bovine serum) was transferred to a corning 15 mL tube. The cryovials were then held under running water until no longer frozen to the wall. The enteroids were transferred to the FWM and centrifuged. These were subsequently suspended in BMM, similar to the above.

### 4.3. Enteroid NEC Induction

For all experiments, the enteroids were mature at the time of experimentation, determined by visualization of budding and ensuring they were 7 days from the last passage. There were, on average, 300 enteroids per well at the time of experimentation. Our lab has previously described a protocol for NEC induction in enteroids in vitro [45]. Enteroids underwent a media change at their normal interval. For cultures undergoing NEC, the media was supplemented with 100 µg/mL lipopolysaccharide (LPS). These were then placed in a modular incubator chamber (MIC—Billings-Rothenberg Inc., Del Mar, CA, USA) and subjected to hypoxia (1% Oxygen). A control group underwent standard media changes and incubation in standard conditions. This protocol was followed for 24 h. This time point has been shown to induce NEC without causing frank necrosis [45,46]. All experiments were performed in triplicate to enhance scientific rigor.

### 4.4. RNA Isolation, Reverse Transcription, and RTqPCR

RNA was isolated from tissue and enteroids. Tissue was processed using the Quiagen^TM^ RNEasy^TM^ Kit (74104; Quiagen, Hilden, Germany), while enteroids were processed using the Invitrogen^TM^ TRIzol^TM^ Reagent (15596026; Life Technologies, Carlsbad, CA, USA). These were performed according to their respective protocols. RNA concentration was quantified using a NanoDrop Lite spectrophotometer (Thermo Scientific, Waltham, MA, USA).

cDNA was generated via reverse transcription to 0.5–2 µg using a high-capacity cDNA reverse transcription kit (Applied Biosystems, #4374966, Waltham, MA, USA). Real-time PCR was then conducted and analyzed using the delta–delta CT method. The genes examined were GPX1, GPX2, and GSS. A list of these genes and their related primers can be found in Table 1. All gene expression was normalized against the housekeeping gene GAPDH. PCR was carried out using the CFX Opus 96 system and iQ SYBR Green Supermix (Biorad, Hercules, CA, USA) with 4 ng of cDNA template and a final primer concentration of 0.5 µM.

### 4.5. NADPH Assay

Enteroids were grown to maturity and subjected to 24 h NEC induction using LPS and hypoxia, as described above. Three wells were pooled into one group to ensure sufficient concentration and detection of NADPH for the assay, with four replicates per group.

NADPH concentration was determined from enteroids via the NADP/NADPH Assay Kit from Abcam (Abcam; abcam65349, Cambridge, UK) after 24 h, as this is our normal timeline for NEC induction. Measurements were taken from a control group. A separate group of enteroids underwent NEC induction as stated above. After 24 h, NADPH levels were rechecked in the original control group and the NEC-induced group. Before performing the assay, the enteroids were subjected to a 10 kD spin column (Abcam; abcam93349, Cambridge, UK) to inactivate enzymes that may rapidly consume NADPH. Reagent preparation, standard preparation, sample preparation, assay procedure, and data calculations were performed according to the manufacturer protocol book provided by Abcam. To determine the NADPH concentration, samples were analyzed via colorimetry and plotted on a line of best fit generated using standards (0–100 pmol).

### 4.6. Illumina RNA-Sequencing Methodology

Stranded RNA-seq libraries were constructed using the NEBNext poly(A) mRNA isolation kit with the IDT’s xGen Broad-range RNA Library Prep Kit and the established protocols (New England Biolabs NEBNext, Ipswich, MA, USA, and Integrated DNA Technologies IDT, Coralville, IA, USA). The library construction was completed using 1 μg of RNA. Each of the libraries was indexed during library construction to multiplex for sequencing. Samples were normalized and pooled onto a 150 paired-end run on Illumina’s NextSeq 2000 Platform (Illumina, San Diego, CA, USA). A heat map of LCN2, NOX01, DUOXA2, and DUOX2 as differentially expressed genes in controls vs. NEC was created.

### 4.7. Immunofluorescence

Intestinal samples were fixed in 10% formaldehyde, embedded in paraffin, and sectioned for histological analysis. These were stained using Immunofluorescence to examine protein expression. Slides were washed twice with warm Phosphate-Buffered Saline (PBS) and then fixed with 2% paraformaldehyde for 1 h at room temperature. Following fixation, slides were washed four times with PBS and then permeabilized with 0.1% Triton in PBS. Slides were blocked with 5% normal goat serum PBS + Tween (PBST, 0.05% Tween, Sigma Aldrich, P9416, St. Louis, MO, USA) for one hour and then incubated overnight with a GPX polyclonal antibody (#GTX100292, GeneTex, Irvine, CA, USA) at a 1:1000 dilution. The following morning, slides were washed with PBST four times and then incubated with a secondary antibody (A11034, Alexa Fluor 488 goat anti-rabbit; Invitrogen, Waltham, MA, USA) for one hour in the dark. After incubation, slides were washed 4 more times with PBS and then mounted on a slide using Fluoroshield with DAPI.

### 4.8. Immunohistochemistry

Tissues were obtained and fixed in 10% neutral buffered formalin, embedded in paraffin, and sectioned in 5 μm sections for routine staining. Sections were deparaffinized, rehydrated, and washed in Tris-Buffered Saline (TBS). Slides were processed for Immunohistochemistry using an IMMPRESS VR Horse Anti-Rabbit IgG polymer kit and a peroxidase Polymer kit (cat# MP-6401, Vector Labs, Newark, CA, USA). Antigen retrieval (pH 6 Citrate Antigen Unmasking Solution (cat# H-3300, Vector Labs Inc., Newark, CA, USA) was accomplished via twenty minutes in a steamer followed by thirty minutes of cooling at room temperature. Sections were treated with a peroxidase blocking reagent (Bloxall, cat# SP-6000, Vector Laboratories, Inc., Newark, CA, USA) to inhibit endogenous peroxidase activity, followed by 2.5% normal horse serum blocking reagent to inhibit nonspecific binding. Appropriate washes were in TBS. Tissue sections were incubated in humidified chambers with Rabbit anti-GPX2 polyclonal antibody (GeneTex, cat# GTX100292, 1:500 dilution, Irvine, CA, USA). Following incubation overnight at 4 °C, sections were washed in TBS, and reagents were applied according to the manufacturer’s directions. Slides were incubated with NovaRed^®^ (Vector Laboratories, Inc., Newark, CA, USA) chromogen for visualization. Counterstaining was carried out with Hematoxylin QS Nuclear Counterstain (Vector laboratories, Newark, CA, USA).

### 4.9. Bathophenathroline Colorimetric Assay

All reagents and standard solutions were made according to the previously established protocol [47]. Throughout the protocol, all samples were handled with plastic tools to prevent iron contamination.

Tissue was cut into 40–50 mg pieces and weights were recorded. These were then placed in a 24-well plate and left to dry in a standard incubator at 65 °C for 48 h. Once completely dry, these were then reweighted and weights recorded. We then proceeded with acid digestion. Samples were moved to 1.5 mL microcentrifuge tubes. Then, 1 mL of the previously made acid mixture was added to each tube. These were placed in a standard incubator at 65 °C for 20 h for digestion. After this, the supernatant was transferred to a new 1.5 mL microcentrifuge tube and stored until use.

For color development, we prepared our working chromogen, acid blanks, and samples according to the previously described protocol [47]. This was performed in a 96-well, clear, round bottom plate (Corning, #07-200-106, Corning, NY, USA). The plate was incubated for 15 min at room temperature. Sample absorbances were then read using a plate reader at 535 nm.

For analysis, we used the following formula to calculate iron levels:

$$\frac{A_{T}-A_{B}}{A_{S}-A_{B}}\times \frac{{Fe}_{S}}{W}\times \frac{\frac{V_{rv}}{V_{smp}}\times V_{f}}{\frac{V_{rv}}{V_{std}}}$$

where:

$A_{T}$: Absorbance of test sample
$A_{B}$: Absorbance of acid blank
$A_{S}$: Absorbance of standard
${Fe}_{S}$: Iron concentration of working iron solution (µg Fe/mL)
W: Weight of dry tissue (g)
$V_{smp}$: Sample volume
$V_{f}$: Final volume of the acid mixture after overnight incubation
$V_{std}$: Volume of iron standard
$V_{rv}$: Final reaction volume

### 4.10. Iron Quantification by ICP-MS

We used ICP-MS to quantify the iron mass in patient samples. We collected approximately 40 to 50 mg of tissue from control and NEC patient groups. We stored samples at −80 °C before tissue digestion. Our digestion process was modeled in prior experiments [48]. After removing tissue samples from storage and allowing samples to warm to room temperature, we added 100 μL of HCl and 400 μL of HNO_3_ to each tissue sample inside a 1.5 mL Eppendorf tube. We transferred the tubes to a warm water bath held at 60 °C for 1 h. After 1 h, we observed the complete dissolution of tissue samples. We removed sample tubes from the warm water bath and allowed the digested tissue solution to cool to room temperature. After mixing tube contents thoroughly, we diluted 125 μL of the digested tissue solution into 4.875 mL of a 2.5 ppb yttrium (Y) solution in nanopure water. Y serves as the internal standard for ICP-MS measurements. After mixing diluted samples thoroughly, we filtered the 5 mL of diluted samples through a sterile 0.22 μm syringe filter and collected the filtrate into 15 mL conical tubes.

We prepared an iron standard curve using the same final acid concentration as the samples (2.0% *v/v* HNO_3_, 0.5% *v/v* HCl). We used an ICP-MS Calibration Standard (10 ppm Fe content) to prepare the standard curve. We quantified the iron mass of each sample using a NexIon 2000 ICP-MS (PerkinElmer, Waltham, MA, USA).

We divided the mass of ^57^Fe by the wet mass of collected tissue to identify the expected average mass of iron (μg) per mass of tissue (mg). We selected ^57^Fe given its relative abundance in biological samples (2.119%), which aligned well with our prepared standard curve. The trends observed for ^57^Fe are expected to remain consistent across all naturally occurring iron isotopes [49].

We analyzed the data using GraphPad PRISM with N = 4 for the control group and N = 4 for the NEC group. We observed no outliers in the data based on Grubb’s outlier test performed on collected measurements.

### 4.11. Lipidomics and Fatty Acid Analyses

Total lipid extraction was performed using a protocol described by Bleigh and Dyer [50]. An aliqout of the total lipid extracts was analyzed by MS/MS analysis of retinal lipids, using 1.0 μmol each of glycerophosphatidylcholine (PC) 14:0/14:0 and glycerophosphatidylethanolamine (PE) 14:0/14:0, 0.33 μmol of glycerophosphatidylserine (PS) 14:0/14:0, and 12.5 nmol of d18:1/12:0 ceramide as internal standards, as we previously described [51]. Quantification of lipid molecular species was performed using the Lipid Mass Spectrum Analysis (LIMSA) software’s peak model fit algorithm (Xcalibur Data System, version 2.2; MassHunter WorkStation, version 10.2) [50].

For fatty acid analyses, 50 nanomoles of pentadecanoic (15:0) and heptadecanoic (17:0) acids were added as internal standards to the total lipid extracts that further underwent acid hydrolysis/methanolysis by heating at 100 degrees Celsius in 16% *v/v* concentrated hydrochloric acid (HCl) in methanol to generate fatty acid methyl esters (FAMEs). FAMEs were extracted in hexane and purified via thin-layer chromatography (TLC) based on a previously described protocol [52]. FAMEs were identified using an Agilent Technologies 7890A gas chromatograph with a 5975C inert XL mass spectrometer detector (Agilent Technologies, Lexington, MA, USA) [51]. FAMEs were quantified using an Agilent Technologies 6890N gas chromatograph with a flame ionization detector [51]. Sample concentrations were determined by comparison to internal standards (15:0 and 17:0). Data are represented as the relative mole percent of each fatty acid.

### 4.12. Statistical Analysis

Results are represented as means, with SEM as error bars unless otherwise reported. Statistical analysis and figures were generated using GraphPad Prism 10.0.0 software. Comparative analyses were run using Student’s *t*-test or One or Two-Way Analysis of Variance (ANOVA), as appropriate. All compared groups had at least three biological replicates and were run in triplicate to improve the rigor of results. Significance was set at a *p*-value of <0.05 prior to analysis.

## 5. Conclusions

This study provides critical insights into the molecular mechanisms underlying NEC, focusing on the roles of oxidative stress, iron dysregulation, and lipid metabolism in the disease. The findings highlight the significant elevation of GPX2 and redox-related gene expression in NEC tissue, indicating an altered oxidative environment that contributes to intestinal injury. Elevated iron levels observed in patients point to a central role in driving oxidative stress through the Fenton reaction and Ferroptotic mechanisms. These radicals cause extensive cellular damage, perpetuating the inflammatory and tissue-degrading processes central to NEC.

In addition to iron accumulation, the study revealed significant lipid remodeling, particularly in PC, PS, and PE species. This suggests that lipid peroxidation and membrane disruption are important features of the disease. The breakdown of phospholipids results in the release of arachidonic acid, which serves as a precursor for pro-inflammatory eicosanoids, such as prostaglandins and leukotrienes. These inflammatory mediators further exacerbate the pathological process, contributing to a sustained and damaging inflammatory response in the intestinal epithelium. The observed alterations in fatty acid profiles, including elevated levels of specific saturated fatty acids, also suggest that lipid metabolism is significantly disrupted in NEC, further amplifying the disease process.

Together, these findings point to ferroptosis as a likely contributor to NEC pathology. The synergy between iron-induced oxidative stress and lipid peroxidation creates a vicious cycle that drives epithelial injury and inflammation. Importantly, this study suggests potential therapeutic targets, such as iron chelation, antioxidant therapies, inhibitors of lipid peroxidation, and ferroptosis inhibitors that may help mitigate the devastating effects of NEC. Further research is needed to explore these pathways and to evaluate their therapeutic potential in clinical settings.

More research is needed to elucidate exactly how the role of NADPH may factor into the pathogenesis of NEC. The paucity of research around the exact mechanisms of oxidative stress and its implications in the pathobiology of NEC remains an interesting domain to explore.

## Figures and Tables

**Table 1:** Forward and Reverse Primer Sequences for GPX1, GPX2, and GSS.

Gene	Forward Primer Sequence	Reverse Primer Sequence
Glutathione Peroxidase 1	5’-CAGTCGGTGTATGCCTTCTCG-3’	5’-GAGGGACGCCACATTCTCG-3’
Glutathione Peroxidase 2	5’-GGTAGATTTCAATACGTTCCGGG-3’	5’-TGACAGTTCTCCTGATGTCCAAA-3’
Glutathione Synthetase	5’-GGGAGCCTCTTGCAGGATAAA-3’	5’-GAATGGGGCATAGCTCACCAC-3’

## Data Availability

The data are not publicly available due to identifying nature of patient data.

## Abbreviations

The following abbreviations are used in this manuscript:

NEC	Necrotizing enterocolitis
NICU	Neonatal intensive care unit
VLBW	Very low birth weight
ROP	Retinopathy of prematurity
BPD	Bronchopulmonary dysplasia
PVL	Periventricular leukomalacia
OS	Oxidative stress
GSH	Glutathione
ROS	Reactive oxygen species
PUFA	Polyunsaturated fatty acid
GPX#	Glutathione peroxidase #
4-HNE	4-hdroxy-2-nonenal
4-HHE	4-hydroxy-2-hexenal
ICP-MS	Inductively coupled plasma mass spectrometry
LCN2	Lipocalin 2
DUOX2	Dual Oxidase 2
DUOXA2	Dual Oxidase maturation factor 2
NOXO1	NADPH oxidase organizer 1
FA	Fatty acids
AA	Arachidonic acid
DHA	Docosahexaenoic acid
IF	Immunofluorescent
IHC	Immunohistochemistry
PC	Phosphatidylcholine
PS	Phosphatidylserine
PE	Phosphatidylethanolamine
IBD	Inflammatory bowel disease
